# Conversion Factors to Compare Serum Concentrations of Anti-HBs, Anti-SARS-CoV-2 and Anti-Tetanus Toxin IgG

**DOI:** 10.3390/antib14030069

**Published:** 2025-08-13

**Authors:** Aurelia Knispel, Christian Jassoy

**Affiliations:** Institute for Medical Microbiology and Virology, University Clinic and Medical Faculty, University of Leipzig, 04103 Leipzig, Germany

**Keywords:** anti-HBs IgG, anti-tetanus toxin IgG, anti-SARS-CoV-2 IgG in serum

## Abstract

**Background**: The concentration of antigen-specific antibodies in serum is usually measured in international units/mL. Therefore, the actual concentration of virus-specific antibodies in sera is unknown. **Objectives**: The aim of the study was to determine conversion factors for concentrations of IgG against hepatitis B surface antigen (HBs), SARS-CoV-2 receptor binding domain (RBD) and nucleoprotein (NP) as well as tetanus toxin (Ttx) in serum and to compare antigen-specific IgG concentrations in serum samples. **Methods**: Absorption equivalence ELISAs were used to determine conversion factors for international units (IU) for anti-HBs, anti-SARS-CoV-2-RBD and NP and for anti-Ttx immunoglobulin G. The antigen-specific IgG concentrations in serum samples were then measured in units/mL and the ratio of IgG concentrations in the sera was determined using the conversion factors. **Results**: One IU of anti-HBs IgG corresponded to 24.4 BAU of anti-CoV-2 RBD IgG, 6.87 BAU of anti-CoV-2 NP and 14 mIU of anti-Ttx IgG. One BAU anti-SARS-CoV-2 NP-specific IgG is equivalent to 3.5 BAU SARS-CoV-2 RBD-specific IgG. Conversion of international units showed that median serum anti-Ttx-IgG concentrations were 50 times higher and anti-CoV-2-RBD-IgG concentrations were 390 times higher than median anti-HBs-IgG concentrations. In addition, after SARS-CoV-2 infection, the concentration of NP-specific IgG in serum was generally higher than that of RBD-specific IgG. **Conclusions**: The study provides conversion factors for serum concentrations of IgG against HBs, SARS-CoV-2 RBD and NP, as well as Ttx-IgG. This offers new insights into serum IgG concentrations and allows conclusions to be drawn about plasma cell pools.

## 1. Introduction

The concentration of antigen-specific antibodies in the serum of patients and, in the case of studies, in test subjects is only measured in mass per volume in exceptional cases. It is usually expressed in units per milliliter. The reason for this is that the specific antibody concentration in the reference sera or plasma, on the basis of which the serum concentrations are measured, has not been determined. Instead, the reference sera and plasma were assigned specific concentrations of antigen-specific immunoglobulin units per milliliter. For example, the WHO International anti-HBs immunoglobulin standard NIBSC code 07/146 has been assigned 1000 international anti-HBs units (IU)/mL [1], the anti-SARS-CoV-2 immunoglobulin standard NIBSC code 20/136 contains 1000 binding antibody units (BAU)/mL [2,3,4]) and the international anti-tetanus toxin immunoglobulin standard NIBSC code 13/240 has been assigned 50.4 international units (IU)/mL [5]. Due to the arbitrary determination of antibody concentrations in the standards, the units cannot be compared with each other. Therefore, the antibody concentrations of different specificities in serum based on units cannot be compared.

There are exceptions to the measurement of antigen-specific antibody concentrations in units. For example, the concentration of antibodies against pneumococcal polysaccharides is usually measured in mass per volume based on the international standard for anti-pneumococcal polysaccharides that has been assigned values in µg/mL [6]. The first anti-pneumococcal immunoglobulin standard has been established with the use of an ELISA which compared antibody concentrations based on absorption equivalence. The ELISA is based on the observation that the strength of the measurement signal in two antibody tests performed in parallel under the same conditions is the same at the same antibody concentrations [6,7,8,9]. This allows to compare the concentration of immunoglobulins against various pneumococcal polysaccharides in sera. The method was also used in the development of immunoglobulin reference sera for meningococci [10].

Against this background, the aim of this study was to determine the conversion factors for anti-HBs, SARS-CoV-2 spike/receptor binding domain (RBD), SARS-CoV-2 nucleoprotein (NP) and Ttx antibody units of the reference sera using an absorption equivalence test in order to compare the concentration of antibodies in the sera.

## 2. Materials and Methods

### 2.1. Antigens, Antibody Standards, Sera

For the ELISAs, high-binding microtiter plates (Greiner Bio-One GmbH, Frickenhausen, Germany) were coated with protein and antibody standard reagents, and serum samples were used. The following proteins were used to coat the plates: recombinant HBs subtype Ad produced in CHO cells (medix Biochemica, https://www.medixbiochemica.com, accessed on 4 June 2025); SARS-CoV-2 Spike-RBD (amino acids 319–519 of the spike ectodomain (Genbank accession no. QHD43416) produced as a fusion protein with a double Strep tag in Expi293 cells; SARS-CoV-2 nucleoprotein (NP) (GenBank accession no. MT007544.1) produced as a fusion protein with *Escherichia coli* maltose-binding protein (MBP) in *E. coli* [11]; Influenza A virus H3N2 (Hong Kong strain 68) nucleoprotein produced as a fusion protein with MBP in *E. coli* [12]; tetanus toxoid (GSK Vaccines GmbH, Marburg, Germany).

The following WHO international standards were used: 2nd International Standard for anti-hepatitis B surface antigen immunoglobulin, NIBSC code 07/146; 1st and 2nd International Standard for SARS-CoV-2 immunoglobulin, NIBSC codes 20/136 and 21/340 [13]; 2nd International Standard for anti-tetanus immunoglobulin, NIBSC code 13/240. The human monoclonal antibody (mAb) SR2-NP66/67 produced in our laboratory was used as the standard for the concentration of influenza nucleoprotein-specific antibodies [14]. The concentration of the mAb in µg/mL was previously determined using an IgG ELISA.

The sera (N = 54) were from adults from a study on the course of the immune response to SARS-CoV-2 [11,15]. The study was reviewed by the Ethics Committee at the University of Leipzig. The subjects gave their informed, written consent.

### 2.2. Absorption Equivalence ELISA

ELISA microtiter plates (Greiner Bio-One, Frickenhausen, Germany) were coated with recombinant HBs, 100 µL/well, 2 µg/mL, SARS-CoV-2 RBD (1 µg/mL) and NP (3 µg/mL), Ttx, (3 µg/mL,) and InfluNP (2–3 µg/mL) in two rows, each. After incubation overnight or longer in the refrigerator, the plates were treated with blocking solution (200 µL/well PBS, 0.05% Tween-20), the antibody standards were diluted in blocking solution and added to the corresponding antigen-coated wells in a two-fold dilution series in duplicates. The final concentrations of the WHO standards were as follows: Anti-HBs 07/164, 12.5–0.2 mIU/mL; Anti-SARS-CoV-2 20/136, 400–6.25 mBAU/mL (RBD); Anti-tetanus toxin 13/240, 160–2.5 µIU/mL; Anti-Influenza NP mAb SR2-NP66/67, 2.5–0.039 ng/mL. Two antigen-coated wells with each of the antigens were spared as blanks. The plates were incubated for one hour at room temperature, then washed, and 100 µL goat anti-human IgG antibody (Jackson Laboratories 109-036-098, diluted 1:2000 in blocking solution) was added to each well. The plates were incubated for another hour at room temperature and washed again. Then, 100 µL TMB substrate (SeramunBlau slow2 85, Seramun Diagnostics GmbH, Heidesee, Germany) was added, and after a few minutes the reaction was stopped by adding 100 µL 0.5 M H_2_SO_4_. Finally, the light absorption of the fluids was measured at a wavelength of 450 nm using a plate photometer. These measurements were carried out four times.

### 2.3. Calculation of the Relative Antigen-Specific Antibody Concentration in the Standards

The optical density (OD) values from the duplicate determinations were averaged and the average blank value was subtracted. The antibody concentrations in the respective units/mL and the OD values were plotted in a coordinate system, the measurement points were connected, and polynomial curve equations were created. The coefficient of determination r^2^ for the curve was determined and the evaluation was optimized so that r^2^ was maximal. In most cases, r^2^ was greater than 0.99. To calculate antibody equivalence concentrations based on the anti-influenza NP mAb SR2-NP66/67, the OD values of each dilution level for the anti-HBs, SARS-CoV-2 RBD and NP and Ttx-Ig standards were entered into the curve equation for the anti-influenza NP IgG standard SR2-NP66/67. This resulted in anti-HBs, SARS-CoV-2 RBD and NP and Ttx equivalence values. To calculate the equivalence values per µg anti-influenza IgG, the anti-HBs, SARS-CoV-2 RBD and NP and Ttx-IgG values in units were divided by the equivalence values in µg. The mean value was calculated from the quotients at the different dilutions of the antibody standards. This corresponds to the ratio of the immunoglobulin units for anti-HBs, SARS-CoV-2 RBD and NP and Ttx-IgG per µg influenza NP-specific IgG of the mAb SR2-NP66/67 (Supplementary Materials Figure S1).

### 2.4. Antibody ELISA with Sera

To compare the antibody concentrations in the sera, standard ELISAs were performed using the same reagents and incubation conditions as the absorption equivalence ELISAs. A microtiter plate was coated with one of the proteins and the plate was blocked with blocking solution. The appropriate antibody standard in a dilution series and the sera in duplicate were applied to the plate. The sera were diluted 1:2000 to 1:100,000 depending on the antigen and antibody concentration. A curve equation was calculated from the concentration and OD values of the antibody standard. The OD values of the sera were entered into the curve equation to determine the antigen-specific IgG concentration of the sera in units/mL. The concentrations were converted to anti-influenza NP IgG equivalents using the conversion factors.

## 3. Results

### 3.1. Equivalence Values and Conversion Factors for IgG Concentrations in Reference Sera

In order to determine equivalence values for different international units, the WHO standards for anti-HBs, anti-SARS-CoV-2 and Ttx immunoglobulins were tested in parallel in absorption equivalence ELISAs. For normalization, the influenza NP-specific mAb SR2-NP66/67 was included as a reference. Subsequently, the number of units of antibody standards corresponding to 1 µg SR2-NP66/67 was calculated by comparing the absorptions. This was performed four times and mean values were determined. The measurements showed that 1 µg of mAb SR2-NP66/67 was equivalent to 4.9 IU anti-HBs IgG, 120.5 BAU SARS-CoV-2 RBD IgG, 34 BAU SARS-CoV-2 NP IgG, and 69 mIU tetanus toxin IgG (Table 1).

The conversion factors between the international antibody units were determined from the equivalence values based on 1 µg of the influenza antibody. For example, 4.9 IU HBs-specific IgG is equivalent to 120 BAU SARS-CoV-2 RBD-specific IgG, 34 BAU SARS-CoV-2 NP-specific IgG, and 69 mIU anti-tetanus toxin IgG. Conversion factors for antibody quantities corresponding to different immunoglobulin units are summarized in Table 2.

### 3.2. Comparison of Antibody Concentrations in Serum Samples

**Figure 1:**
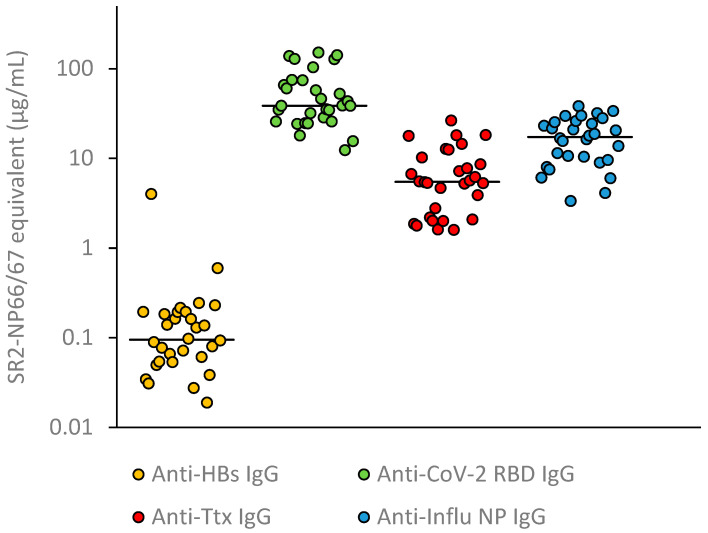
Concentration of antigen-specific IgG antibodies relative to the anti-influenza NP reference standard SR2-NP66/67 in 30 sera from SARS-CoV-2 vaccinated individuals. Lines show the medians. The medians of the four antibody concentrations were statistically significantly different from each other (Wilcoxon rank test, *p* < 0.0001).

In order to compare the concentration of IgG against HBs, SARS-CoV-2 RBD and tetanus toxin in sera, the antigen-specific IgG concentrations in the corresponding international units were first determined using ELISA. The values were then normalized to µg SR2-NP66/67 equivalents using equivalence values and displayed in a scatter plot. The concentrations of anti-HBs, SARS-CoV-2 RBD and Ttx IgG in the sera differed significantly. The anti-HBs IgG concentrations were the lowest. The anti-Ttx IgG concentrations were approximately 50-fold higher and the SARS-CoV-2 RBD-specific IgG concentrations were 390-fold higher than the median anti-HBs IgG concentration (Figure 1).

### 3.3. NP- and RBD IgG Concentrations in Sera from Infected and Vaccinated Individuals

In the absorption equivalence ELISA, the equivalence value of RBD- and NP-specific BAU was 120.5 to 34. This means that approximately 3.5 BAU of RBD-specific IgG corresponded to 1 BAU of SARS-CoV-2 NP-specific IgG. This conversion factor was used to compare the SARS-CoV-2 RBD- and NP-specific IgG concentrations in sera from SARS-CoV-2-infected individuals 2–10 weeks and 6–7 months post-infection and 7 months post vaccination. According to the BAU values, the sera almost consistently appeared to have a higher concentration of RBD-specific antibodies. However, after conversion, the NP IgG concentration was found to be higher than the RBD-specific IgG concentration in most cases during the first months after infection (Table 3).

## 4. Discussion

The concentrations of anti-HBs, SARS-CoV-2-RBD and Ttx-specific antibodies in serum are markers for the immune response to vaccination against hepatitis B, COVID-19 and tetanus. They are not measured in mass per volume, but in units/mL. However, this does not indicate the concentration ratio between the antibodies. To determine this, absorption equivalence ELISAs were performed with the international immunoglobulin standards for anti-HBs, SARS-CoV-2 and Ttx-IgG. The antibody concentrations were normalized to the anti-influenza NP concentration of a mAb, and the relative antigen-specific IgG concentrations were determined. This resulted in equivalence values and conversion factors for the international units. These were used to compare the concentrations of HBs, SARS-CoV-2 RBD and NP, and Ttx-specific IgG in serum samples.

Absorption equivalence ELISAs are based on the observation that the concentration of antibodies against different antigens is the same when the measurement signal in the test is the same [6,7]. To ensure equivalence, the measurements with the antibody standards were performed in parallel on the same microtiter plate, and the incubation times, the concentration of the secondary antibody, and the development time were kept the same. Repeated measurements yielded similar conversion factors. The reference value µg mAb SR2-NP66/67 was a tool for making the measured values comparable with different antibody standards. One of the other units could also have been used as the basis for the calculations and would have produced similar results.

The measurement of antibodies against HBs, SARS-CoV-2 RBD and Ttx in serum showed significant differences between antigen-specific IgG concentrations. The high antibody concentration against SARS-CoV-2 RBD is likely due in part to the fact that the subjects had received a booster vaccination against SARS-CoV-2 six months prior to blood collection. The lowest serum concentration was measured for anti-HBs IgG. The mean concentration of Ttx-specific IgG was a good 50 times higher, and the CoV-2 RBD-specific IgG concentration was an additional 7.5 times higher. The determination of the relative antibody concentration provides information about the relative amount of antibody-secreting cells in the body. For example, the low anti-HBs IgG concentration in serum indicates that the pool of long-lived anti-HBs IgG-producing plasma cells in the bone marrow is significantly smaller than the pool of anti-Ttx-secreting cells.

In sera from SARS-CoV-2-infected individuals, the virus spike RBD-specific IgG concentration in BAU/mL was usually higher than the NP-specific IgG concentration after infection. Only when the concentrations were converted using the conversion factor did the NP-specific IgG concentrations in serum prove to be higher than the RBD-specific IgG concentrations. Thus, comparing the actual antigen-specific antibody concentrations in serum provides deeper insight into the antibody response to the infection.

The conversion factors can be used to compare antigen-specific immunoglobulin concentrations in serum. For example, it can be investigated whether the minimum immunoglobulin concentration required to protect against disease is the same for different infections. Previous studies have shown that an antibody concentration of 10 mIU/mL anti-HBs IgG, measured 1–2 months after vaccination, provides lasting protection [16]. Similarly, a concentration of 0.1–0.2 IU/mL anti-Ttx IgG in ELISA is considered protective [17]. No threshold value has been established for SARS-CoV-2, but in a large European efficacy study for COVID-19 vaccines, a vaccine efficacy of 80% was achieved with 506 BAU/mL anti-SARS-CoV-2-RBD [18]. Converting the concentrations according to Table 2, 10 mIU anti-HBs IgG corresponds to 0.14 mIU anti-Ttx IgG and 0.24 BAU SARS-CoV-2 RBD IgG. This means that a significantly lower specific IgG concentration is associated with protection against hepatitis B than with protection against tetanus or COVID-19. Similarly, 0.1–0.2 IU anti-Ttx IgG corresponds to 57–114 BAU SARS-CoV-2 RBD Ig indicating that the concentration of protective anti-Ttx IgG is lower than that required for 80% protection against COVID-19.

The conversion factors determined in the study can possibly be used to achieve a better understanding of the IgG response in clinical situations. For example, the concentration of anti-HBs, Ttx and other specific IgG in serum is occasionally measured in units/mL in children with antibody production deficits to monitor the course of the immune response after vaccination [19]. The conversion factors can be used to compare antibody concentrations for different vaccine proteins and, indirectly, the size of the plasma cell pools in the children.

The absorption equivalence ELISA is a rarely performed procedure. When performed carefully, the test is very accurate. The coefficient of variation in the equivalence factors in the four measurements from Table 1 was 7–10%. The absorption equivalence ELISAs performed by Quataert and colleagues using a pneumococcal reference serum showed a similarly low variation in the measured values [6,9]. To check whether the values deviate systematically, we determined the conversion factors in a second procedure. In this procedure, we used memory B cell ELISpot and ELISA to measure the antigen-specific IgG release per antibody-secreting cell [20]. We obtained similar conversion factors (manuscript in preparation).

## 5. Conclusions

In summary, conversion factors for anti-HBs, anti-SARS-CoV-2 and anti-Ttx IgG concentrations in international units were determined using absorption equivalence tests. This allowed the antibody concentrations against different viral proteins and vaccine antigens in the serum to be compared with each other. After SARS-CoV-2 infection, the NP-specific IgG response was stronger than the RBD-specific antibody response. The concentration of IgG against different vaccine antigens and the minimum protective IgG concentrations differed significantly. Since the antibodies are produced by plasma cells, this also provides an insight into the size of the plasma cell pools.

## Figures and Tables

**Table 1:** Equivalence values of antibody standards relative to µg of mAb SR2-NP66/67.

	Anti-HBs IgG 07/164 ^1^ (IU)	Anti-CoV-2 RBD IgG 20/136 ^1^ (BAU)	Anti-CoV-2 NP IgG 20/136 ^1^ (BAU)	Anti-Ttx IgG 13/240 ^1^ (mIU/mL)
Measurement	/µg SR2-NP66/67 equivalent			
1	5.18	125	33.2	63.5
2	4.7	110	31.6	68.2
3	5.43	130	32.7	78.7
4	4.45	117	38.4	65.4
Mean	4.9	120.5	34.0	69.0
Standard deviation	0.44	8.81	3.03	6.78
Coefficient of Variation	0.090	0.073	0.089	0.098

^1^ NIBSC code of international standard.

**Table 2:** Conversion factors for international units for anti-HBs, anti-SARS-CoV-2 RBD and NP, and anti-Ttx-IgG.

Ratio ^1^	Anti-			
Anti-	HBs (IU)	CoV-2 RBD (BAU)	CoV-2 NP (BAU)	Ttx (mIU)
HBs (IU)		24.5	6.87	14.0
CoV-2 RBD (BAU)	0.041 ^1^		0.28	0.57
CoV-2 NP (BAU)	0.145	3.55		2.03
Ttx (mIU)	0.072	1.75	0.49	

^1^ Units in columns per unit in rows, for instance 0.041 IU anti-HBs IgG per 1 BAU Anti-CoV-2 RBD IgG.

**Table 3:** Ratio of RBD- and NP-specific IgG concentrations in serum samples after SARS-CoV-2 infection and vaccination.

Sample ID	Time Point	RBD BAU/mL	NP BAU/mL	RBD BAU/ NP BAU	RBD IgG/ NP IgG
CoV-001	Early ^1^	556	269	2.1	0.59
	6 months ^2^	123	35	3.5	0.99
	Vaccinated ^3^	2486	18	135.2	38.53
CoV-003	early	465	306	1.5	0.43
	6 months	234	149	1.6	0.45
	vaccinated	557	87	6.4	1.83
CoV-006	early	402	163	2.5	0.70
	6 months	107	48	2.2	0.64
	vaccinated	1443	8	179	50.94
CV220/003	early	45	23	2.0	0.56
	6 months	11	4	2.8	0.78
	vaccinated	10	2	5.6	1.59
CV220/008	early	104	45	2.3	0.66
	6 months	50	33	1.5	0.44
	vaccinated	420	8	53.1	15.14
CV220/010	early	3208	1500	2.1	0.61
	6 months	320	204	1.6	0.45
	vaccinated	6842	97	70.5	20.08
CV220/024	early	121	83	1.5	0.42
	6 months	193	31	6.3	1.80
	vaccinated	6313	10	656.4	187.07
CV220/035	early	94	467	0.2	0.06
	6 months	101	73	1.4	0.40
	vaccinated	2210	12	186.1	53.03

^1^ early: 2–10 weeks after infection; ^2^ 6 months: 6–7 months after infection; ^3^ vaccinated: 7 months after vaccination.

## Data Availability

Further data will be made available by the corresponding author upon request.

## Supplementary Materials

The following supporting information can be downloaded at: https://www.mdpi.com/article/10.3390/antib14030069/s1, Figure S1: Example for calculations of an absorption equivalence ELISA.

## Abbreviations

The following abbreviations are used in this manuscript:

SARS-CoV-2	Severe acute respiratory distress syndrome coronavirus-2
RBD	Receptor binding domain
NP	Nucleoprotein
BAU	Binding antibody units
