# Supplementary material for: Conversion Factors to Compare Serum Concentrations of Anti-HBs, Anti-SARS-CoV-2 and Anti-Tetanus Toxin IgG
Source: Antibodies (Basel). 2025 Aug 13;14(3):69. doi: 10.3390/antib14030069 (PMC12371921; doi:10.3390/antib14030069)
Supplement: Supplementary file 1 [file antibodies-14-00069-s001.zip › antibodies-3713156-supplementary.pdf]

## Conversion factors to compare serum concentrations of anti-HBs, anti-SARS CoV-2 and anti-tetanus toxin IgG

Aurelia Knispel and Christian Jassoy

**Supplementary Material**

**Suppl. Figure S1: Example for calculations of an absorption equivalence ELISA. A)** OD values for influenza NP-specific mAb SR2-NP66/67 on influenza virus NP and SARS CoV-2 Ig standard 20/136 on SARS CoV-2 RBD in dilution series; **B)** Antibody standard curves, curve equations, and coefficient of determination for the standard curves; **C)** Influenza IgG equivalence antibody concentration and calculation of the ratio of BAU to µg SR2-NP66/67.

**A)**

| Influenza NP-IgG | | SARS CoV-2 RBD-IgG | |
|---|---|---|---|
| **SR2-NP66/67(ng/ml)** | **OD** | **WHO 20/136 (mBAU/ml)** | **OD** |
| **1,25** | 1,39 | **200** | 2,00 |
| **0,63** | 0,80 | **100** | 1,13 |
| **0,31** | 0,44 | **50** | 0,63 |
| **0,16** | 0,24 | **25** | 0,31 |
| **0,08** | 0,13 | **12,5** | 0,16 |
| **0,04** | 0,07 | **6,25** | 0,08 |

**B)**

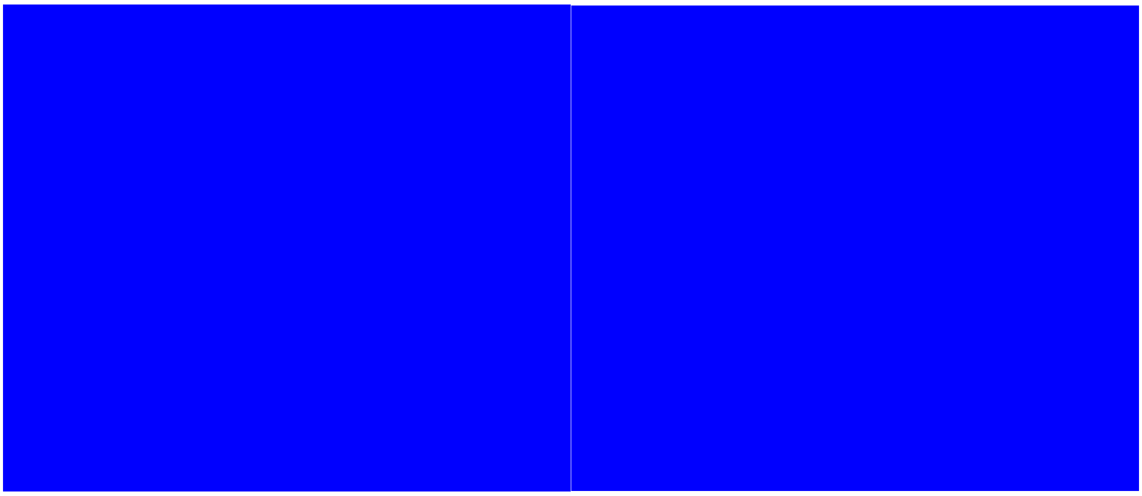

**C)**

| SARS CoV-2 RBD-IgG mBAU/ml | Influenza NP equivalent ng/ml SR2-NP66/67 | RBD/Influenza NP IgG ratio |
|---|---|---|
| 200,0 | 2,04 | 98,0 |
| 99,8 | 0,97 | 103,4 |
| 50,7 | 0,47 | 108,6 |
| 24,2 | 0,21 | 114,8 |
| 12,5 | 0,10 | 122,9 |
| **Average** | | 110 |
